# Above- and belowground herbivory jointly impact defense and seed dispersal traits in *Taraxacum officinale*

**DOI:** 10.1002/ece3.1172

**Published:** 2014-07-31

**Authors:** Eduardo de la Peña, Dries Bonte

**Affiliations:** 1 Terrestrial Ecology Unit (TEREC), Department of Biology, Faculty of Sciences, GhentUniversityK.L. Ledeganckstraat 35, Gent, 9000, Belgium; 2 Instituto de Hortofruticultura Subtropical y Mediterránea “La Mayora”, Universidad de Málaga – Consejo Superior de Investigaciones CientíficasAlgarrobo-Costa, Málaga, E-29750, Spain

**Keywords:** Maternal effects, plant defense, resistance, tolerance, trade-off, trichomes

## Abstract

Plants are able to cope with herbivores by inducing defensive traits or growth responses that allow them to reduce or avoid the impact of herbivores. Since above- and belowground herbivores differ substantially in life-history traits, for example feeding types, and their spatial distribution, it is likely that they induce different responses in plants. Moreover, strong interactive effects on defense and plant growth are expected when above- and belowground herbivores are jointly present. The strengths and directions of these responses have been scarcely addressed in the literature. Using *Taraxacum officinale,* the root-feeding nematode *Meloidogyne hapla* and the locust *Schistocerca gregaria* as a model species, we examined to what degree above- and belowground herbivory affect (1) plant growth responses, (2) the induction of plant defensive traits, that is, leaf trichomes, and (3) changes in dispersal-related seed traits and seed germination. We compared the performance of plants originating from different populations to address whether plant responses are conserved across putative different genotypes. Overall, aboveground herbivory resulted in increased plant biomass. Root herbivory had no effect on plant growth. Plants exposed to the two herbivores showed fewer leaf trichomes than plants challenged only by one herbivore and consequently experienced greater aboveground herbivory. In addition, herbivory had effects that reached beyond the individual plant by modifying seed morphology, producing seeds with longer pappus, and germination success.

## Introduction

Herbivory affects plant populations and community structure through detrimental effects on plant performance and fitness (Strauss et al. 2002; Fornoni 2011). Therefore, plants have evolved traits that enable them to cope with this stress. Induced plant defenses comprise either chemical responses, through the production of secondary metabolites, or physical structures such as spines, thorns, and trichomes, which act as deterrents against herbivores (Dicke and van Loon 2000; Hanley et al. 2007; Kaplan et al. 2008a,b). Another mechanism to avoid herbivores is changing plant architecture, allowing a spatial escape from herbivore pressure. Spatial escape responses occur by modification of root/shoot biomass ratios (Peinetti et al. 2001; Stevens et al. 2008) or by allocating biomass to enemy-free plant parts, as happens in clonal plants (D'Hertefeldt and Jonsdottir 1999; Benot et al. 2009; de la Peña and Bonte 2011). In nonclonal plants, induction of seed dispersal may allow a temporal escape of the offspring from herbivores (Wender et al. 2005; Fresnillo and Ehlers 2008; Poethke et al. 2010).

Defense and dispersal are typically expressed as plastic responses conditional on the experienced environment, and are known to have a strong genetic component as expressed by *genotype* × *environment* (G × E) reaction norms (Agrawal et al. 2002; Holeski et al. 2010). Becuase herbivory is heterogeneous in space and time, its impact on plant fitness is likely to differ according to the herbivore life-history traits and the spatial distribution of involved species (Agrawal 2007; de la Peña et al. 2011). Over the last decade, the interaction of herbivores occurring above- and belowground on ecological dynamics has been extensively studied (van Dam and Heil 2011). However, while most studies to date focused on the mechanisms of above- and belowground interactions (Heil 2011; van Dam and Heil 2011; Kaplan et al. 2008a,b; Bezemer and van Dam 2005), genetic differentiation in resistance to herbivory and induced defenses have received less attention.

Defensive traits comprise, among many other, morphological structures that constrain herbivore activity. Leaf toughness, trichomes, thorns, and spines may be induced to act as a mechanical barrier against herbivore feeding (Hanley et al. 2007). Trichomes act as mechanical barriers against herbivores, and their function in this sense is twofold: (1) they obstruct herbivore movement and feeding, and (2) some trichomes may harbor chemicals that act as deterrents to herbivorous insects (Tuberville et al. 1996; Valverde et al. 2001). Leaf pubescence, that is, trichome density, has been reported to be plastic in response to changes in several abiotic environmental conditions (Roy et al. 1999; Perez-Estrada et al. 2000; Sam et al. 2000), but also in response to aboveground herbivory (Pullin and Gilbert 1989; Traw and Dawson 2002). Whether this aboveground physical defense can be induced by belowground herbivory, as is the case for chemical defense responses, is currently unknown.

Increased dispersal is also expected to be selected under stressful conditions as a fitness maximizing strategy (Levin et al. 2003; Bonte et al. 2012). Because dispersal capacity in plants is with some exceptions restricted to seeds; dispersal is under maternal control. It is therefore expected to increase when conditions experienced by the mother deteriorate, as it is the case with local herbivory (Ronce and Olivieri 1997; Donohue and Schmitt 1998; Nathan and Casagrandi 2004). In addition, different herbivores (above- and belowground herbivores) with different life-history traits are expected to exert differential selection pressures on their host plants (de la Peña et al. 2011) and as such, to induce different dispersal responses.

In wind-dispersing plants, seed dispersal distance is predicted to depend on the seed's terminal velocity as determined by seed weight and dispersal structures like pappus hairs or seed wings (Tackenberg et al. 2003). In addition, Thomson et al. (2011), demonstrated the importance of plant height, or the height of the flowering scapus, on dispersal distance, with seeds released from taller plants ending up at further distances. Experimental work showing plasticity in seed dispersal traits in relation to herbivory is almost nonexistent (but see Donohue 1998). Moreover, whether plastic responses in both defense and dispersal can be induced by herbivores feeding on belowground plant parts remains understudied.

The objective of this study was to investigate to what degree above- and belowground herbivory affects the direction and strength of (1) plant growth responses, (2) induction of plant defensive traits, that is, leaf trichomes, and (3) changes in dispersal-related seed traits (i.e., scapus height, seed mass, seed morphology) and seed germination. In all cases, we compared plants coming from different genotypes of dandelion *Taraxacum officinale* F.H. Wigg. This species was selected because (1) it has seeds dispersed by wind; (2) seed production and seed morphology are plastic traits influenced by different environmental conditions; (3) it is a host for both above- and belowground herbivores; and (4) the selected individuals were sampled from apomictic populations (Weeda et al. 1999), so that offspring from one mother-plant is genetically identical (i.e., obtaining different isolines) and in consequence, performance in a common garden is driven by maternal inheritance.

Aboveground herbivory was exerted by the locust *Schistocerca gregaria* (Insecta: Orthoptera). This species is a widespread generalist herbivore and is known for being particularly devastating to crops. As with all Acridid species, it feeds on plants by chewing on their leaves. As root herbivore, we use the northern root-knot nematode *Meloidogyne hapla* (Nematoda: Heteroderidae). This nematode species penetrates into plant roots, where it triggers a physiological response that leads to root modification. Gravid females of *M. hapla* stay inside the roots, which result in the coating of such sessile females with root tissue; these infection symptoms are commonly known as *root-knots*. As for other root-feeding nematodes, one of the common effects of nematode attack is the disruption of plant hydric balance, resulting in plant growth similar to that of water-stressed plants (Moens and Perry 2009).

We hypothesized that root-feeding nematodes would affect the physiological balance of the host plant, inducing leaf trichomes (as in water-stressed plants) and by doing so, modifying interaction with foliar feeders. According to the stress hypothesis (White 1993), we expected a positive effect of root-feeding nematodes on aboveground herbivores, that is, locusts would preferably feed on stressed plants. In relation to seed dispersal traits, we hypothesized that seed traits related to enhanced dispersal would be expressed (e.g., higher pappus/seed mass ratio) when aboveground herbivory is present; mainly because they have, based on biomass consumption, a larger impact on plants than root herbivores.

## Materials and Methods

### Collection of plant material

Seeds from one plant from six different populations were collected in natural or urban grasslands in Belgium and Germany (Table S1). Because experimental plants showed distinct variation in leaf morphology (Fig. S1a), and because of the considerable distance between them even within the same geographic area (i.e., within Belgium or Germany), we regard them as different genotypes.

### Preparation of experimental pots

Seeds were kept in a cool, dry place until the experiments were set up. The seeds used to generate plants for all experiments were surface-sterilized by submersion for 20 sec in a 4% household bleach solution. They were subjected to 10 washes with distilled water and subsequently submerged for 40 sec in a 10% ethanol solution, followed by 10 additional washes with distilled water. After surface sterilization, seeds were germinated in plastic incubators (17.5 cm × 13 cm × 6 cm), filled with sterilized river white sand (±2 cm thick), and moistened with demineralized water to soil saturation. Between 75 and 100 seeds per genotype were placed in each incubator. The seed containers were set under 36-watt lights, with a 16-h light/8-h dark photoperiod regime, at 23 ± 1°C and 35% relative humidity. After germination, seedlings grew for 2 weeks before being transplanted to experimental pots. 1.4-L pots were filled with a mixture of white river sand and potting soil at a proportion of three parts sand to one part potting soil. One seedling was transferred to each pot. Plants were grown for a total of 10 months.

### Experimental setup

The experiment compares the performance of plants, bred from a single mother-plant from six populations of *T. officinale* (i.e., three Belgian and three German genotypes, see supplementary material for more information), in four treatments: (1) control plants, those growing in sterilized soil; (2) plants inoculated with root-feeding nematodes; (3) plants treated with locusts; and (4) plants treated with both root-feeding nematodes and locusts. We established eight replicates for each treatment and genotype. Because of the apomictic reproduction of the species, we consider all offspring from one mother-plant as being genetically identical (isolines) and refer to these as genotypes. The experiment took place in a growth chamber, where plants received artificial light. Plants were watered every other day until soil saturation and were fertilized once a month with 20 mL of a commercial fertilizer (20:20:20 NPK).

### Inoculation of nematodes in experimental plants

Five weeks after transplantation, the plants were inoculated with nematodes. A 0.5-cm-wide and 5-cm-deep hole was perforated next to the root system of each plant. Then, using a 1-mL glass pipette, 100 lab-reared juveniles (J2) of *Meloidogyne hapla,* suspended in 3 mL of demineralized water, were injected into the pot (Southy 1986). The same procedure was applied, but with a previously microwaved solution of nematodes, to the remaining treatments.

### Treatment of plants with locusts

From preliminary experiments, we knew that the selected aboveground herbivore feeds avidly on *T. officinale*; therefore, a constant exposure to the locusts would result in complete defoliation of plants and eventually death, so we applied the herbivores twice during the course of the experiment. Prior to application of the locusts, plants were placed randomly in a growth chamber. Afterward, we released 20 adult locusts (scapula size of 8.33 ± 0.51 mm, mean ± SD). The animals were kept grazing for two different periods of 7 days. In case, a plant was flowering at that particular moment, we protected the flowers by covering them with a veil until the locusts were removed. Herbivory occurred in February and April of 2009. After 7 days, the herbivores were removed from the experimental setup and we estimated the percentage of herbivory (Fig. S2a–c). Herbivory was measured by assessing the percentage of leaf area consumed by the locust (so called in percentage of defoliation).

### Plant harvest

From the initial 192 pots, we collected data from 162 pots, because some plants died in the course of the 10-month experiment. At harvest time (the first week of June 2009), shoot and root biomass were assessed. The incidence of root herbivores was measured by counting the number of root-knots (i.e., mature females) in the roots. For the assessment of trichomes (Fig. S1b), a leaf was taken from each plant, and the number of trichomes in the transect of 1 cm was counted under a binocular microscope. The transect was always located at the point equidistant to the tip and base of the leaf. The trichomes were counted along the one-centimeter transect from the leaf nerve outwards. We additionally measured the length of each flowering scapus for further analysis.

### Seed morphology and germination of F2

Seeds were collected during the course of the experiment and stored in paper envelopes at room temperature. We obtained seeds for all the compared treatments from only two genotypes (i.e., Citadel [Belgium-B2] and FS5 [Germany-G3]), from the other genotypes either plants did not flower or only from one of the treatments did. We counted the number of seeds produced by each flower and selected five random seeds for measurement of mass and length of three randomly selected pappus hairs per seed. The length of three pappus hairs was determined after scanning a seed placed on millimeter paper with a household scanner (HP) at a resolution of 96 dpi. Seeds were weighted on a balance with 10^–4^ g precision.

Twenty-five seeds from each flower head were put in a Petri plate lined with filter paper to quantitate germination rate. The Petri dishes were put on shelves under the previously explained light regime. Filter paper was moistened daily with distilled water, and the germinated seeds were counted every other day. Seeds were considered germinated when the primary root or cotyledons could be observed. After 30 days, there was no further germination and the experiment was stopped.

### Statistical analysis

Error structure of all variables was checked prior to statistical testing with a Kolmogorov–Smirnov test and by plotting residuals against their linear predictors. According to the error structure of the response variables (i.e., normal, Poisson or binomial), we used linear mixed models (i.e., normal error structure) or generalized linear mixed Models (i.e., binomial or Poisson error structure) using the SAS (9.4) procedures Proc Mixed or Proc GLIMMIX (with quasi-likelihood estimation of parameters). In all cases, treatment (with four levels i.e., control, nematodes, locust, nematode + locusts) was modeled as fixed factor; genotype and genotype × treatment interactions were modeled as random effects to correct for similarities in responses among plants coming from the same origin. For seed-related traits, date of flowering (and consequently of seed production) was also included as a random factor in the model due to the fact that plants flowered several times during the course of the experiment. Differences among the different treatments were assessed with Tukey's post hoc HSD test. Due to the presence of random effects, effective degrees of freedom were computed through the Satterthwaites's approximation of SAS (Verbeke and Molenberghs 2009). With this general schema, we addressed differences in root, shoot, percentage of defoliation, seed mass, and scapus and pappus length with linear mixed models. The mortality of experimental plants and germination of F2 seeds (both binomial variables), root-knots per plant, and number of seeds and trichomes (all with Poisson error structure) were modeled using a GLMM.

## Results

### Plant traits

**Figure 1:**
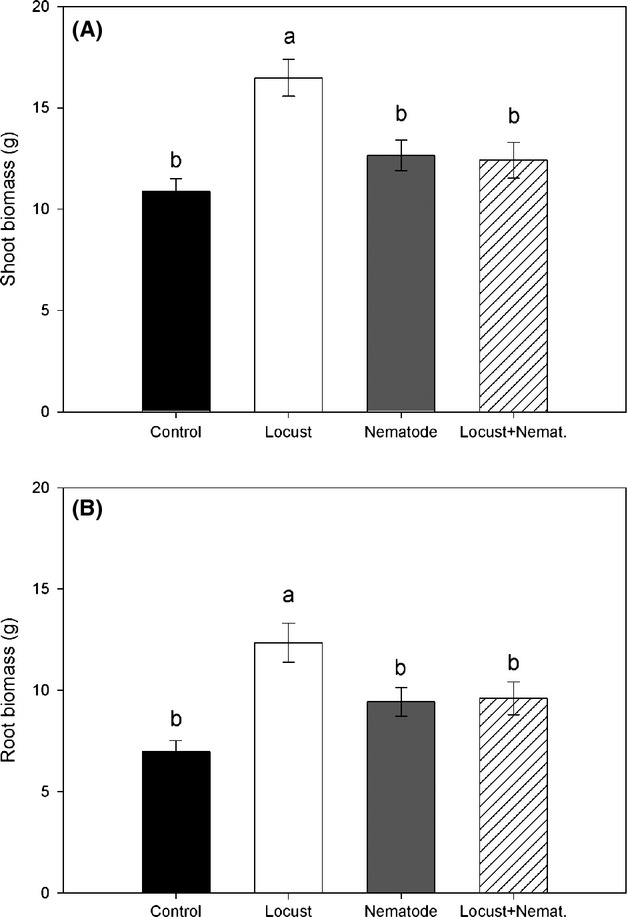
Shoot (A) and root biomass (B) (mean ± SE) of *Taraxacum officinale*. Different letters indicate significant differences according to treatment (i.e., control, locust, nematodes, and nematodes + locusts), according to Tukey's post hoc test (*P* ≤ 0.05).

Plant mortality occurred randomly across treatments and genotypes (Treatment *F*_188, 3_ = 0.91, *P* = 0.43; genotype *Z* = 0.99; *P* = 0.16; genotype × Treatment *Z* = 0, *P* = 0.98). Differences according to treatment on shoot biomass, and root biomass were detected (LMM results see Table 1). In this sense, locust herbivory resulted in plants significantly larger, that is, with more biomass, than plants from the other three treatments (Fig. 1A). The same pattern was observed in root biomass, with a significant increase in plants exposed only to locusts (Fig. 1B). Root herbivory per se did not have an impact on plant biomass; however, the presence of root herbivores seems to inhibit the compensatory biomass production observed in plants exposed solely to locusts (Fig. 1A).

**Table 1:** Statistics of: (1) the linear mixed model (LMM) testing treatment (i.e., control, nematodes, locust, nematode + locust), modeled as fixed factor, on root, shoot biomass and percentage of defoliation; genotype and genotype × treatment were included as random effects; (2) the generalized linear mixed model (GLMM) testing the same fixed and random factors on no. of trichomes and no. of root-feeders (*n* = 162)

	LMM						GLMM			
		
	Root biomass		Shoot biomass		% of defoliation		No. of trichomes		No. of root-feeders	
					
	*F*	*P*	*F*	*P*	*F*	*P*	*F*	*P*	*F*	*P*
Fixed effects										
Treatment	4.97	**0.012**	14.56	**<0.001**	25.13	**<0.001**	5.16	**0.01**	3.70	0.07

	*Z*	*P*	*Z*	*P*	*Z*	*P*	*Z*	*P*	*Z*	*P*
Random effects										
Genotype	0.84	0.20	1.50	0.06	0.82	0.20	1.42	0.08	–	–
Genotype × Treatment	1.30	0.09	–	–	0.66	0.25	0.27	0.39	1.36	0.08

Figures in bold indicate significant differences (*P* ≤ 0.05).

### Impact of above- and belowground herbivory

**Figure 2:**
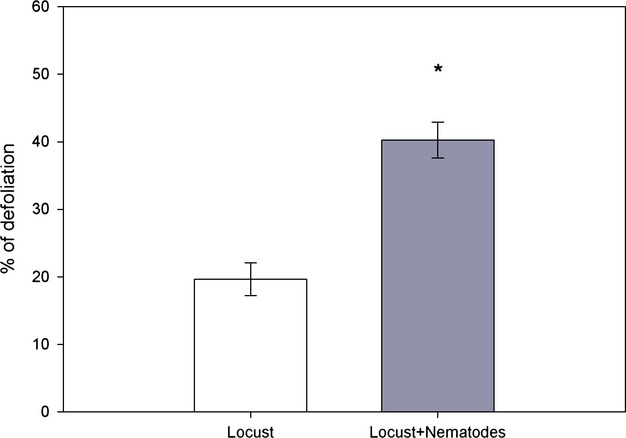
Percentage of defoliation (mean ± SE) on leaves of *Taraxacum officinale* exposed to the locust *Schistocerca gregaria*, or to the locust and the root herbivore *Meloidogyne hapla*. Asterisks indicate significant differences according to Tukey's post hoc test (*P* ≤ 0.05) between the two treatments.

The presence of root herbivores increased the percentage of defoliation. In other words, plants exposed to nematodes and locusts suffered more leaf loss than plants without nematodes (Fig 2). The effect of root herbivores on percentage of defoliation was consistent and in the same direction for all genotypes compared as random factors were highly insignificant (Table 1). Besides the drastic initial impact from defoliation of locusts on plants, leaf herbivory resulted on the long term in larger plants. With a marginal *P* = 0.07, locust herbivory yielded plants with less root-knots than locust-free plants (Table 1). Again, the substantial (though only marginally significant) G × E interaction term (Table 1) suggests that not all genotypes reacted in the same direction and strength when exposed to both herbivores.

### Plant defense – trichomes

**Figure 3:**
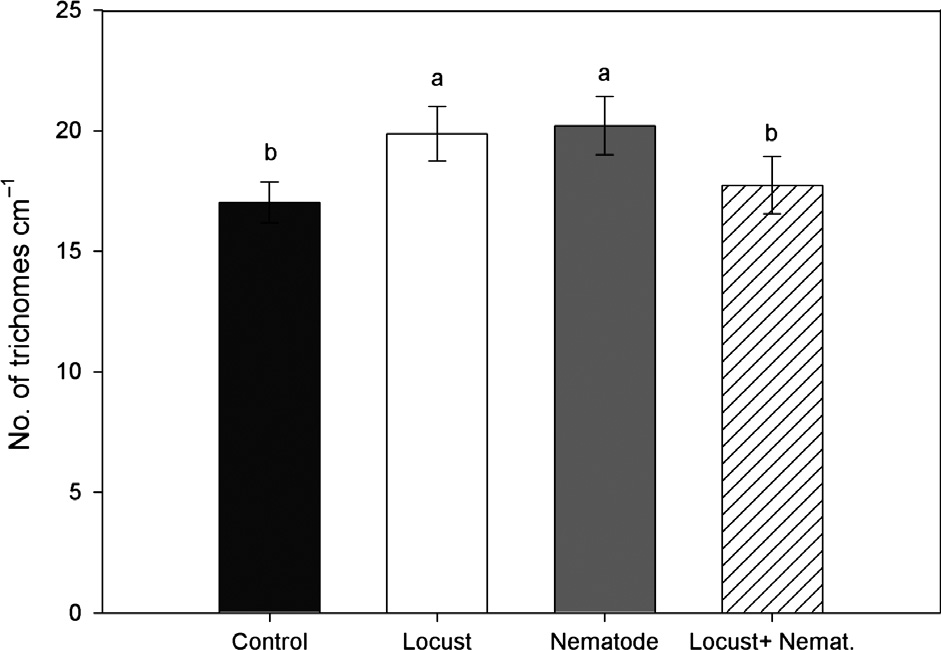
Number of trichomes on leaves (mean ± SE) of *Taraxacum officinale* according treatment (i.e., control, locust, nematode, locust + nematode). Different letters indicate significant differences according to Tukey's post hoc test (*P* ≤ 0.05).

The presence of herbivores, regardless of whether they appeared above- or belowground, resulted in a higher trichome density (Fig. 3). However, the combination of nematodes and locusts caused a suppression of trichome production, reaching values similar to control plants (Fig. 3).

### Dispersal-related traits and F2 germination

**Figure 4:**
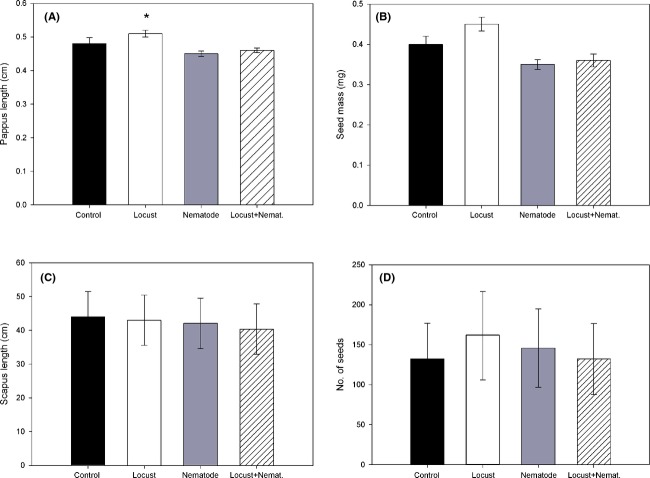
Variation in seed traits (mean ± SE) of *Taraxacum officinale*: (A) pappus length, (B) seed mass, (C) length of flowering stalk (scapus length), and (D) number of seeds per plant. An asterisk indicates a significant difference of a particular treatment with respect to the others according to Tukey's post hoc test (*P* ≤ 0.05).

Treatment had a significant effect on pappus length. For the genotypes used in the experiment, plants only exposed to locusts increased pappus length (Fig. 4A). When plants where exposed to nematodes or to both herbivores, pappus length was similar to that of control plants. For the other seed dispersal traits (i.e., scapus length, seed mass, or no. of seeds), no effect of treatment was detected (Table 2, Fig. 4B–D).

**Table 2:** Statistics of: (1) the linear mixed model (LMM) testing treatment (i.e., control, nematodes, locust, nematode + locust), modeled as fixed factor, on scapus and pappus length; genotype, date of flowering, and the genotype × treatment interaction as random effects; (2) the generalized linear mixed model (GLMM) testing the same fixed and random factors on no. of seeds and percentage of germination rate (*n* = 111)

	GLMM				LMM					
		
	No. of seeds		Germination rate		Seed mass		Pappus length		Scapus length	
					
	*F*	*P*	*F*	*P*	*F*	*P*	*F*	*P*	*F*	*P*
Fixed effects										
Treatment	1.47	0.38	16.70	**0.02**	4.31	0.33	5.81	**0.001**	1.04	0.51

	*Z*	*P*	*Z*	*P*	*Z*	*P*	*Z*	*P*	*Z*	*P*
Random effects										
Genotype	0.63	0.26	–	–	0.39	0.34	0.69	0.24	0.72	0.23
Genotype × Treatment	1.14	0.12	0.75	0.22	0.24	0.40	–	–	0.69	0.24
Date	4.04	0.0001	2.28	**0.01**	–	–	–	–	–	–

Figures in bold indicate significant differences (*P* ≤ 0.05).

**Figure 5:**
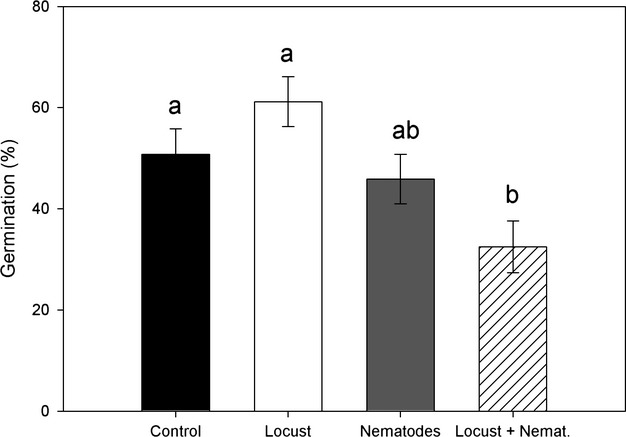
Percentage of seed germination [(mean ± SE) of *Taraxacum officinale* (F2) according to treatment. Different letters indicate significant differences according to Tukey's post hoc test (*P* ≤ 0.05).

Germination rate was negatively affected by the common effect of locusts and nematodes (Table 2, Fig. 5). In this case, the date of flowering also showed a strong effect on the germination of the seeds (Table 2).

## Discussion

The study of interactions between herbivores occurring above- and belowground has received considerable attention over the last decade. Research has primarily focused on the signs and directions of the interaction and, to a lesser extent, on the mechanisms involved (Bezemer and van Dam 2005; Kaplan et al. 2008a,b; Heil 2011; Van Dam and Heil 2011). In very few instances, the interaction between herbivores has been investigated using different plant genotypes of one plant species, and not full-sibs like our reared *T. officinale* lines (Schweitzer et al. 2008; Vandegehuchte et al. 2011; Genung et al. 2012). Moreover, whether herbivores occurring at different plant parts are able to affect seed dispersal traits has, to our knowledge, never been studied experimentally. As we know from other species, both defense and dispersal are not fixed among populations and are subjected to selection by genotype × environment interactions (G × E). Given that herbivore occurrence is heterogeneous in space and time, herbivore impact on plants differs according to the life-history traits and spatial distribution of the herbivores (Agrawal 2007; de la Peña et al. 2011). As in our experiment, by means of a reaction-norm approach, that is, using offspring originating from genetically distinct mother-plants, we show that trichome production has a genetic background and therefore, the outcome of the interactions depends of the genotype considered. Moreover, it is strongly affected by above- and belowground herbivores.

Above- and belowground herbivory in this study had contrasting effects on plant performance. While aboveground herbivory yielded plants with the largest biomass, plants treated only with nematodes or exposed to both herbivores yielded biomasses as large as that of the control treatment, indicating that locust grazing resulted in an over-compensatory production of plant biomass. Such over-compensatory response was consistent among the six tested genotypes and highlights the resilient character of dandelions to aboveground herbivory. Compensatory responses have been reported in different plant species and are considered to be an adaptive trait to cope with herbivore pressure (Lowenberg 1994; Paige 1999; Strauss and Agrawal 1999; Olejniczak 2011). In contrast, belowground herbivory, that is, in nematode-treated plants, resulted in the suppression of the over-compensatory growth response (i.e., showing full-compensation, a biomass similar to control values).

Root-feeding nematodes, and root-knot nematodes in particular, are well-known organisms in agricultural systems that are able to induce physiological stress in a multitude of crops (Moens and Perry 2009). From a functional point of view, nematodes are not only able to elicit local and systemic defensive responses (Kaplan et al. 2008a,b), but their infection often results in structural modifications oriented to reduce water loss, for example closing of leaf stomata or decreasing photosynthetic activity, and prevent further damage (Belair 2005; Volkmar 1991). We focused on the induction of leaf trichomes because it has been shown to be a plastic trait under different environmental factors (Pullin and Gilbert 1989; Perez-Estrada et al. 2000). We hypothesized that root-feeding nematodes could modify production of leaf trichomes and by doing so, the interaction of plants and herbivores occurring in the foliage. Because belowground herbivory also inhibits the compensatory growth response (see above), trade-offs among different resistance strategies seem to be prevalent in the studied system (Herms and Mattson 1992; Ballhorn et al. 2008). Exposure of plants to herbivores in this experiment, regardless of type, resulted in a higher number of trichomes than in control plants (see Fig. 2); however, when plants were subjected to both herbivores at the same time, leaf trichomes showed similar values to the control plants. This suggests that the combined action of above- and belowground herbivory during plant growth levies costs on resistance induction, which results in increased aboveground herbivory. However, this interpretation, linking number of trichomes and herbivory, should be taken with caution as we only measured the number of trichomes at harvest. Moreover, other mechanisms ruling the interaction between the two herbivore groups cannot be excluded (Rudenskaya et al. 1998; Vellend et al. 2009). *Taraxacum officinale* as member of the Asteraceae, is a highly lactiferous species (Wahler et al. 2009). Latex contains multiple secondary metabolites, and in the particular case of this species, it is very rich in polyphenols which, as seen for other species, are crucial in the defense against pathogens and insect herbivores (Vaughn and Duke 1984). Moreover, *T. officinale* produces glandular trichomes that are usually involved in the production, emission, and modulation of many secondary metabolites (Glas et al. 2012). In order to have a better understanding of defense responses in this species, the production and content of glandular trichomes and the trade-offs of these traits should be further studied.

Treatments with both herbivores resulted in a lower number of root-knots, indicating a negative effect of locusts on the multiplication of nematodes. Presence of nematodes resulted in locusts with greater size, that is, thorax length and as such, indicates asymmetric interaction strengths between the above- and belowground herbivores. Interactions between above- and belowground herbivores are highly diverse and thus difficult to predict (Moran and Whitham 1990; Tindall and Stout 2001; Blossey and Hunt-Joshi 2003; Staley et al. 2007; Kaplan et al. 2008a,b). Positive effects of root-feeders on leaf herbivores have been explained through inducement of water stress, causing an increase in the amount of soluble nitrogen in leaves (White 1984; Masters 1995), or by damaging secondary metabolite production sites in the roots (Kaplan et al. 2008a,b). Although we did not assess the physiological status of our plants, our results are in line with these earlier findings indicating that increased plant stress resulted in more plant consumption and ultimately a better growth in locusts (Bernays and Lewis 1986).

Aside from the induced defenses, we observed significant effects of aboveground herbivory on dispersal-related seed traits, that is, pappus length. While scapus length and seed mass were not impacted by herbivory, increased pappus length relative to seed mass was a consistent response in plants exposed to locusts. Such plastic response in seed morphology decreases the seed's terminal velocity and therefore, increases potential dispersal distance (Soons et al. 2004; Tackenberg et al. 2003). This indicates that plants are able to respond in a very short period to herbivory by adjusting pappus length, thereby inducing bet-hedging strategies in the distribution of offspring (Muller-Landau 2003). Aboveground herbivory did consequently induced plastic responses in seed traits that represent a shift toward a colonizing syndrome, expected to evolve under environmental condition characterized by low dispersal costs and high levels of environmental stochasticity (Clobert et al. 2001; Jakobsson and Eriksson 2003; Bonte and de la Pena 2009). However, given the limited number of isolines that flowered under all four treatments, we cannot be conclusive to which degree these responses are quantitatively variable among genotypes. While we controlled herbivory to such levels that all plants survived, such a strategy is likely at the cost of reaching maturity under natural conditions.

Transgenerational effects of herbivores on plants have been observed for different species suggesting that such plant responses may be adaptive and as such amplify or constrain natural selection on the progeny (Agrawal 2001; Latzel et al. 2014). Transgenerational effects can be mediated by differences in storage reserves, toxins, hormones (in the seed embryo), or by epigenetic mechanisms. Interestingly, although plants are exposed to herbivores occurring both above- and belowground, studies on maternal effects have mainly focused on aboveground herbivores. Here, we show that the joint action of root and shoot herbivores have a strong effect on the germination of *T. officinale*. We cannot point forward any mechanism, as we did not measure any parameter at seed level, but seed biomass does not seem to be involved as it did not differ between the experimental treatments. Nonetheless, the results here presented open, interesting venues for future research; not only for the species in consideration, but for species from other natural systems challenged by herbivores feeding above- and belowground.

Our study shows that plant growth and changes in seed morphology as response to above- and belowground herbivory are consistent across the *T. officinale* genotypes compared. It also shows that both root and shoot herbivores are able to induce leaf trichomes; however, the response in this case is genotype dependent which strongly suggests that root herbivores play an important role in eco-evolutionary processes for this species. We demonstrate experimentally that above- and belowground herbivores may have effects that go beyond the individual plant by modifying seed morphology and germination success. Finally, our study shows that although root herbivores do not have a direct effect on plant growth and seed morphology, their presence has important modulatory effects for those traits when plants are also exposed to herbivores aboveground.

## Supporting Information

**Table S1.** Geographical and habitat information of *Taraxacum officinale* populations used in the experiment. **Figure S1.** (a) Comparison of leaf morphology of *Taraxacum officinale* populations used in the experiment; (b) detail of leaf trichomes. **Figure S2.** Damage caused by the locust *Schistocerca gregaria* in *Taraxacum officinale*. **Figure S3.** (a) No. of root-knots (mean ± SE), *Meloidogyne hapla*, in roots of *Taraxacum officinale*. (b) Thorax length (mean ± SE) for *Schistocerca gregaria*. An asterisk indicate significant differences after a post-hoc Tukey test (*P* ≤ 0.05).
