# Supplementary material for: Above- and belowground herbivory jointly impact defense and seed dispersal traits in Taraxacum officinale
Source: Ecol Evol. 2014 Jul 31;4(16):3309–19. doi: 10.1002/ece3.1172 (PMC4222217; doi:10.1002/ece3.1172)

**Supplementary material**

| **Table 1** Geographical and habitat information of *Taraxacum officinale* populations used in the experiment. Belowground herbivory indicates the presence of *M. hapla* (root-knot nematode) in the location. Aboveground herbivory indicates the presence of locusts in the sampled location. | | | | | |
| --- | --- | --- | --- | --- | --- |
| Location name | Code | Coordinates | Location | Habitat | Herbivory |
| Watersportbaan  (UGent) | B1 | N 51° 2’ 53.74’’  W 3°41’ 14.91’’ | Ghent, Belgium | Urban-grassland | Belowground |
| Citadelpark | B2 | N 51° 2’ 10.75’’  W 3°43’6.95’’ | Ghent, Belgium | Urban-grassland | Belowground |
| Ringvaart | B3 | N 51° 1’ 17.62  W 3°44’43.51’’ | Ghent, Belgium | Semi-urban grassland | Above- and belowground |
| Rauhenebrach (FS1) | G1 | N49°55’16.3’’  E10°33’6’’ | Germany | Grassland | Above- and belowground |
| Rauhenebrach (FS3) | G2 | N49°45’32’’  E10°21’5.7’’ | Germany | Grassland | Above- and belowground |
| Rauhenebrach (FS5) | G3 | N48°45’16’’  E10°18’5’’ | Germany | Grassland | Above- and belowground |

**Fig. 1** (a) Comparison of leaf morphology of *Taraxacum officinale* populations used in the experiment; (b) detail of leaf trichomes.


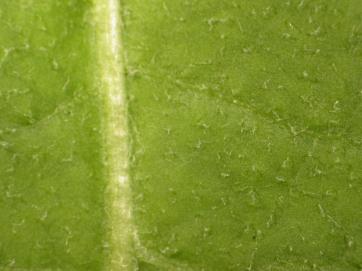

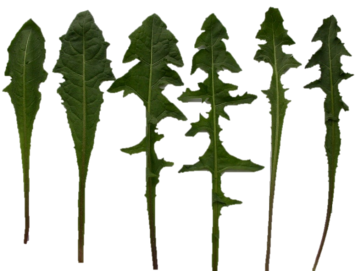


**a**

**b**

**Fig. 2**  Damage caused by the locust *Schistocerca gregaria* in *Taraxacum officinale.* (a) Plant with little or no signs of herbivory; (b) partial defoliation along the leaf nerve; (c) complete defoliation.


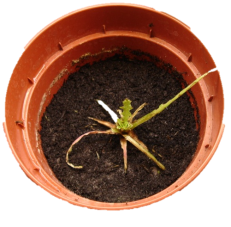

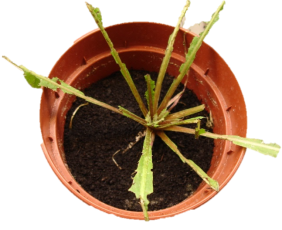

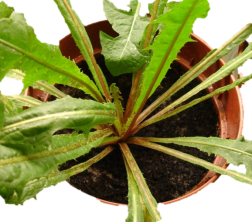


**a**

**b**

**c**

**Fig. 4** (a) No. of root-knots (mean ± SE), *Meloidogyne hapla,*  in roots of *Taraxacum officinale*. (b) Thorax length (mean ± SE) for *Schistocerca gregaria* An asterisk indicate significant differences after a post-hoc Tukey test (p ≤ 0.05).

**a**

**b**


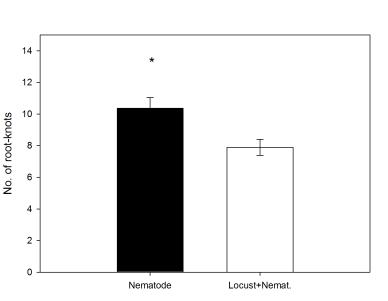


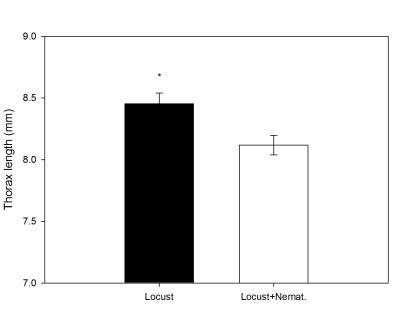

Supplement: Supplementary file 1 [file ece30004-3309-sd1.docx]
